# Prognostic Value of Neutrophil-to-Lymphocyte Ratio in Patients with Acute Decompensated Heart Failure: A Meta-Analysis

**DOI:** 10.3390/jcm13051212

**Published:** 2024-02-21

**Authors:** Song Peng Ang, Jia Ee Chia, Vikash Jaiswal, Muhammad Hanif, Jose Iglesias

**Affiliations:** 1 Department of Internal Medicine, Rutgers Health/Community Medical Center, Toms River, NJ 08755, USA; songpeng.ang@rwjbh.org; 2 Department of Internal Medicine, Texas Tech University Health Science Center, El Paso, TX 79905, USA; jchia@ttuhsc.edu; 3 Department of Internal Medicine, Larkin Community Hospital, South Miami, FL 33143, USA; vikash29jaxy@gmail.com; 4 Department of Internal Medicine, Suny Upstate Medical University, Syracuse, NY 13210, USA; hanifafridi273@gmail.com; 5 Department of Internal Medicine, Hackensack Meridian School of Medicine, Nutley, NJ 07110, USA

**Keywords:** acute congestive heart failure, inflammation, neutrophile lymphocyte ratio, mortality metaanalysis

## Abstract

**Background:** Inflammation plays a pivotal role in the pathogenesis of both acute and chronic heart failure. Recent studies showed that the neutrophil-to-lymphocyte ratio (NLR) could be related to adverse outcomes in patients with cardiovascular diseases. We sought to evaluate whether NLR could predict mortality in patients with acute heart failure by means of a meta-analysis. **Methods:** A comprehensive literature search was performed in PubMed, Embase, and Cochrane databases through January 2023 for studies evaluating the association of NLR with mortality in patients with acute heart failure. Primary outcomes were in-hospital mortality and long-term all-cause mortality. Endpoints were pooled using a random-effects DerSimonian-and-Laird model and were expressed as a hazard ratio (HR) or mean difference (MD) with their corresponding 95% confidence intervals. **Results:** A total of 15 studies with 15,995 patients with acute heart failure were included in the final study. Stratifying patients based on a cut-off NLR, we found that high NLR was associated with a significantly higher in-hospital mortality [HR 1.54, 95% CI (1.18–2.00), *p* < 0.001] and long-term all-cause mortality [HR 1.61, 95% CI (1.40–1.86), *p* < 0.001] compared to the low-NLR group. Comparing the highest against the lowest NLR quartile, it was shown that patients in the highest NLR quartile has a significantly heightened risk of long-term all-cause mortality [HR 1.77, 95% CI (1.38–2.26), *p* < 0.001] compared to that of lowest NLR quartile. However, the risks of in-hospital mortality were compared between both quartiles of patients [HR 1.78, 95% CI (0.91–3.47), *p* = 0.09]. Lastly, NLR values were significantly elevated among non-survivors compared to survivors during index hospitalization [MD 5.07, 95% CI (3.34–6.80), *p* < 0.001] and during the follow-up period [MD 1.06, 95% CI (0.54–1.57), *p* < 0.001]. **Conclusions:** Elevated NLR was associated with an increased risk of short- and long-term mortality and could be a useful tool or incorporated in the risk stratification in patients with acute heart failure.

## 1. Introduction

Myocardial injury from ischemic heart disease, as well as mechanical stresses such as pressure volume overload, results in the discharging of damage-associated molecular patterns from injured or damaged cells into the extracellular space [1]. These patterns are danger signals that interact with the intracellular and membrane-associated pattern recognition receptors of neighboring cells, resulting in activation of the innate immune system and the release of proinflammatory cytokines [2,3,4]. The resulting recruitment of neutrophils and cells of the macrophages and monocyte lines causes a para-inflammatory response necessary for removing cellular debris, proper tissue repair, and adaptation [1,2]. However, this initial compensatory inflammatory response may lead to dysregulated chronic inflammation, an acceleration of atherosclerosis, and the worsening of congestive heart failure [1,3,4].

Evidence within the last several decades has demonstrated the role of inflammation in contributing to the progression of cardiovascular disease, and in essence, it can be considered a non-traditional risk factor in disease progression and clinical outcomes [1,2,3,5,6]. Increases in circulating pro-inflammatory cytokines such as Il-6 and TNF-α correlate with the severity of cardiovascular disease [1,2,3,4,5,7]. Elevated proinflammatory cytokines such as IL-6 and TNF-α are also found in patients with congestive heart failure and are not only indicative of the severity of the disease but may also have deleterious effects on cardiac myocytes and vascular endothelial cells [1,8,9]. In particular, there has been an increased focus on the correlation of proinflammatory cytokines and other circulating biomarkers with the severity of congestive heart failure (CHF) and clinical outcomes. Evidence suggests that other biomarkers such as C-reactive protein (CRP) and the neutrophil lymphocyte ratio (NLR) correlate with elevations in circulating pro-inflammatory cytokines and can be implemented as surrogate measures for acute and chronic inflammation [1,3,6,10,11,12]. In addition, the NLR is indicative of the balance between the innate and adaptive immune system. In particular, both CRP and NLR correlate with elevations in circulating proinflammatory cytokines, the severity of congestive heart failure, and prognosis. The neutrophil lymphocyte ratio is a simple ratio obtained from a routine hemogram (CBC) and is rapidly and easily obtainable. Previous meta-analyses of the prognostic value of the NLR ratio have been mixed due to heterogeneity in patient population and the sizes of the studies reviewed [13]. With this in mind, we conducted a meta-analysis of the association of NLR and adverse outcomes among patients with acutely decompensated CHF.

## 2. Methods

This systematic review was reported following the Cochrane and PRISMA (Preferred Reporting Items for Systematic Review and Meta-analysis) 2020 guidelines [14]. A pre-specified study protocol has been registered in the PROSPERO (CRD42023394287).

### 2.1. Search Strategy and Study Selection

We conducted a systematic search in PubMed, Embase, and Cochrane Central for articles from their inception to January 2023 using the following keywords: “neutrophil”, “lymphocyte”, “neutrophil lymphocyte ratio”, “NLR”, or “heart failure”. Two authors (SPA and JEC) performed the title and abstract screening and subsequently reviewed the articles for eligibility. Studies that were included had all the following parameters: (i) patients with acute heart failure; (ii) studies with patients >18 years; (iii) studies reporting at least one of the desired outcomes; and (iv) cohort studies or randomized controlled trials. We excluded literature or systematic reviews, letters, animal studies, and studies with patients <18 years of age. Any discrepancies during the screening and selection process were resolved by the third author (VJ).

### 2.2. Data Extraction and Quality Assessment of Included Studies 

Study data were independently extracted by two authors (VJ and JEC), and any discrepancies were checked against the original article and resolved by a third author (SPA). Data from the eligible studies, such as demographic, study design, follow-up, and clinical outcomes among both groups of patients, were extracted to a spreadsheet by two authors (SPA and JEC). The primary outcomes of this meta-analysis were in-hospital mortality and all-cause mortality. Secondary outcomes include the difference in the NLR values between survivors and non-survivors of acute heart failure. Clinical outcomes derived from two arms of differential NLR values (high vs. low based on a specified cut-off value of NLR) were extracted. In addition, the baseline NLR values for studies comparing survivors and non-survivors of acute heart failure during index hospitalization and during the follow-up period were obtained. The methodological quality of each study was independently evaluated by two authors (SPA and JEC) using the Newcastle–Ottawa Scale for observational studies, with any disagreements being resolved by a third author (JI). 

### 2.3. Statistical Analysis 

We conducted a conventional meta-analysis for primary outcomes and adopted the DerSimonian-and-Laird random-effect model for the study variations [15]. The outcomes were analysed in 3 cohorts. First, we measured the outcomes between cohorts of high and low NLR. Second, we compared the outcomes between cohorts of highest and lowest quartiles. Lastly, we analyzed the crude NLR values between survivors and non-survivors among patients with acute heart failure during index hospitalization and a follow-up period. In our analysis of categorical outcomes, we selected the most-adjusted form of effect size, where available, for pooled analysis. The outcomes were reported as pooled hazard ratio (HR) for categorical data and mean difference (MD) for continuous data, along with their corresponding 95% confidence interval (95% CI). In addition, we assessed the between-study heterogeneity using the Higgins I-square (I^2^) test, with I^2^ values <75% considered mild–moderate and >75% considered high. Sensitivity analysis was performed using a leave-one-out method for outcomes with at least 5 studies to test the robustness of the primary analysis. Assessment of publication bias was performed for outcomes with at least 5 studies using funnel plots as well as Egger’s regression test. All statistical work, including analysis and graphical illustrations, was conducted using STATA (version 17.0, StataCorp (College Station, TX, USA)). 

## 3. Results

### 3.1. Study Selection 

Preliminary search through the databases yielded 1651 articles, of which 393 studies were excluded after the removal of duplicates. A total of 1182 studies were further excluded after screening the titles and abstracts. A full-text review was conducted for the remaining 76 articles, of which 61 studies were excluded for the following reasons: wrong patient population; insufficient data; lack of relevant outcomes; review articles or case series. 

Finally, a total of 15 studies met the eligibility criteria and were included in the meta-analysis [11,12,16,17,18,19,20,21,22,23,24,25,26,27,28]. The Preferred Reporting Items for Systematic Reviews and Meta-Analyses (PRISMA) flow diagram is depicted in Supplementary Figure S1.

### 3.2. Baseline Characteristics of Studies

All 15 studies were observational cohort studies, with 9 studies [11,12,17,18,19,20,22,23,24] being retrospective studies while the remaining 6 studies [16,21,25,26,27,28] were prospective in nature. A total of 15,995 patients with acute heart failure were studied. Characteristics of the included studies are depicted in Table 1.

### 3.3. Quality Assessment

The methodological quality of the analyzed studies was evaluated using the Newcastle–Ottawa Scale, with the outcomes quantified in terms of stars and converted to the Agency for Healthcare Research and Quality (AHRQ) criteria of “good”, “fair”, or “poor”. The quality scores of the included studies varied, with a range between seven to nine stars. Among these, eight studies were classified as “good”, one study was deemed “fair”, and the remaining six were considered “poor” in quality. A significant factor contributing to the lower quality ratings was the inadequate adjustment for confounders, which could potentially influence the effect size of interest. Notably, nine out of fifteen studies reported effect sizes adjusted for relevant covariates, with the specifics of these adjustments and the covariates involved detailed in Supplementary Table S2. Details regarding the quality evaluation of each study are available in Supplementary Table S1.

### 3.4. High vs. Low NLR 

**Figure 1:**
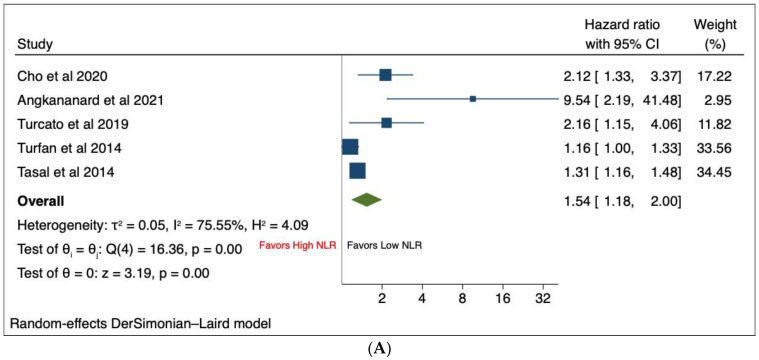

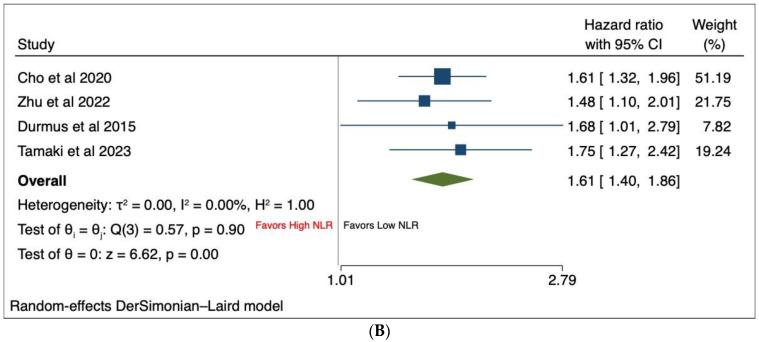
Forest plots of outcomes including (**A**) in-hospital mortality and (**B**) long-term all-cause mortality based on high vs. low NLR.

Eight studies [16,17,19,21,24,25,27,28] compared the association between NLR and outcomes based on a cut-off value specified in their respective studies. Five studies [16,17,19,24,25] reported on in-hospital mortality, while four studies [21,25,27,28] reported on long-term all-cause mortality. The results of the meta-analysis showed that high NLR was associated with a significantly higher risk of in-hospital mortality [HR 1.54, 95% CI (1.18–2.00), *p* < 0.001, I^2^ = 75.6%] (Figure 1A). Furthermore, the high-NLR group has a significantly higher risk of long-term all-cause mortality compared to the low-NLR group [HR 1.61, 95% CI (1.40–1.86), *p* < 0.001, I^2^ = 0%] (Figure 1B). Sensitivity analysis showed that for in-hospital mortality, the results remained largely unaltered and consistent with the primary analysis, showing the robustness of analysis (Supplementary Figure S2). There was evidence of funnel plot asymmetry for in-hospital mortality, suggestive of publication bias (Supplementary Figure S3). Further adjustment of publication bias using the trim-and-fill method showed that results remained significant and that high NLR remained significantly associated with an increased risk of in-hospital mortality [HR 1.37, 95% CI (1.04–1.82)] (Supplementary Figure S4). Sensitivity analysis and assessment of publication bias were not performed for all-cause mortality given the small number of studies.

### 3.5. Highest vs. Lowest NLR Quartile

**Figure 2:**
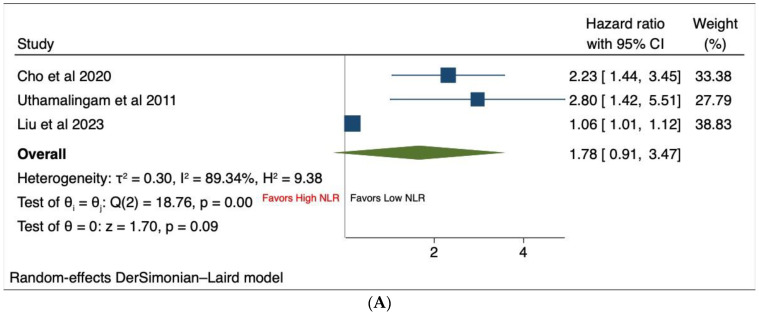

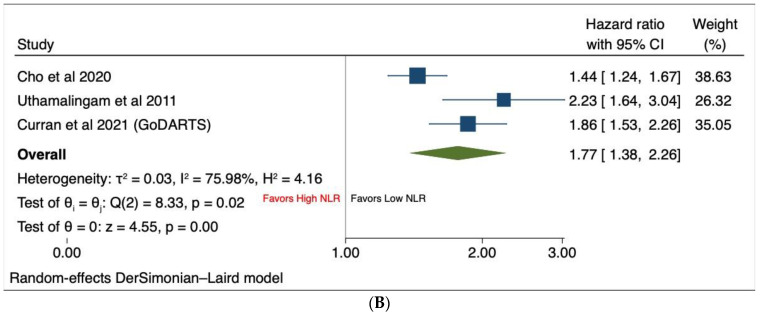
Forest plots of outcomes including (**A**) in-hospital mortality and (**B**) long-term all-cause mortality based on highest vs. lowest NLR quartiles.

Four studies compared the outcomes for patients with acute heart failure based on their NLR quartiles, with the highest quartile being compared to the lowest NLR quartile [11,12,25,26]. Of these, three studies [12,25,26] provided data on in-hospital mortality and three studies [11,25,26] reported on long-term all-cause mortality. The results of the meta-analysis revealed that there was no significant difference in terms of in-hospital mortality between the two groups of the patients [HR 1.78, 95% CI (0.91–3.47), *p* = 0.09, I^2^ = 89.3%] (Figure 2A). However, the risk of long-term all-cause mortality was significantly elevated in patients among the highest NLR quartile compared to those in the lowest NLR quartile [HR 1.77, 95% CI (1.38–2.26), *p* < 0.001, I^2^ = 76%] (Figure 2B). Sensitivity analyses and evaluation of publication bias were not carried out due to the limited number of studies included. 

### 3.6. Non-Survivors vs. Survivors

**Figure 3:**
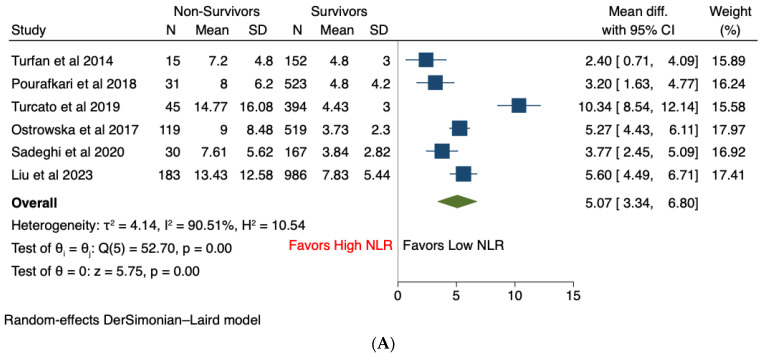

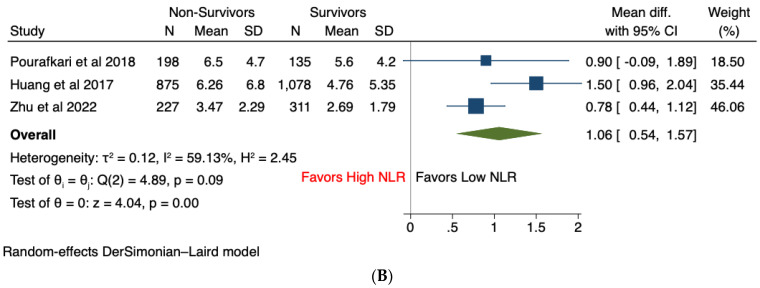
Forest plots of differences in NLR values among non-survivors compared to survivors: (**A**) during index hospitalization; (**B**) during follow-up period.

Eight studies stratified patients with acute heart failure into a non-survivors group and a survivors group [12,16,18,19,20,21,22,23]. Among these, six studies [12,16,18,19,22,23] reported the NLR values during index hospitalization, while three studies [18,20,21] provided the NLR values between survivors and non-survivors during the follow-up period. During index hospitalization, non-survivors were found to have significantly higher NLR values compared to survivors [MD 5.07, 95% CI (3.34–6.80), *p* < 0.001, I^2^ = 90.5%] (Figure 3A). Likewise, during the follow-up period, NLR was significantly elevated among non-survivors compared to survivors of acute heart failure [MD 1.06, 95% CI (0.54–1.57), *p* < 0.001, I^2^ = 59%] (Figure 3B). Both effect sizes remained significant and unaltered after sensitivity analysis using the leave-one-out method, confirming the robustness of the analysis (Supplementary Figure S5). The funnel plot was symmetrical for mean difference during index hospitalization, suggesting no evidence of publication bias (Supplementary Figure S6). Given the limited number of studies, sensitivity analysis and assessment of publication bias were not performed for mean difference during the follow-up period. 

## 4. Discussion

To the best of our knowledge, this is the most comprehensive meta-analysis to evaluate the prognostic value of NLR on patients with acute heart failure. Using the cut-off model, we demonstrated that higher NLR was associated with a significantly higher risk of both in-hospital mortality and long-term all-cause mortality. Using another model of comparing highest and lowest quartiles, higher NLR continued to have a significantly increased risk of long-term all-cause mortality. However, there was a trend of increased risk of in-hospital mortality when comparing the highest against the lowest quartile, but it ultimately did not reach statistical significance, likely due to the paucity of studies included in that analysis (*n* = three studies). In general, our study echoed the findings of a prior meta-analysis conducted by Wang et al., who assessed the value of NLR in both acute and patients with chronic heart failure, whereby elevated NLR was associated with increased mortality and higher risk of renal dysfunction [13].

Elevated neutrophil-to-lymphocyte ratio (NLR) is not only associated with worse outcomes in patients with heart failure (both acute and chronic); its predictive value has been studied in other cardiovascular pathologies in the literature. A meta-analysis conducted by Zhang et al. to evaluate the prognostic value of NLR in patients with acute ST elevated myocardial infarction (STEMI) after percutaneous coronary intervention (PCI) and showed that raised NLR was correlated with significantly increased in-hospital cardiac mortality, major cardiovascular events (MACE), advanced heart failure, and overall mortality in such patients [29]. Similarly, NLR was also found significantly higher in the aortic disease group and was associated with increased mortality in such patients [30]. Another study conducted by Kim et al. found that elevated NLR (≥2.15) was prospectively and significantly associated with all-cause mortality, coronary heart disease, and heart failure [31].

Inflammation plays a vital role in the pathogenesis and progression of heart failure, promoting fibrosis and remodeling through different mechanisms [32]. Inflammatory markers such as C-reactive protein (CRP), markers derived from complete blood counts such as WBC, neutrophils, lymphocytes, monocytes, and platelets have been shown to play an important role in immune responses, and changes in their levels are associated with cardiovascular events and all-cause mortality in patients with heart failure [33,34,35]. Various possible mechanisms have been explained in the literature that explain why elevated NLR is associated with worse outcomes in patients with heart failure. Arruda-Olsan et al. conducted a community-based study to evaluate the effect of neutrophilia in acute myocardial infarction patients and revealed that neutrophilia is independently associated with increased occurrence of heart failure and overall mortality in acute MI patients [36]. Activated neutrophils release various proteolytic enzymes, i.e., acid phosphatase, myeloperoxidase, and elastase, the enzymes which result in the destruction of cardiomyocytes [37,38]. These inflammatory markers, i.e., elastase and myeloperoxidase, were found to be significantly higher in patients with heart failure, showing that inflammation is potentially associated with the pathogenesis of heart failure and increased mortality in such patients [39,40]. On the other hand, lymphocytes are associated with the regulation of immune system pathways, and lymphocytopenia has been found to be associated with increased mortality in heart failure in various studies [41,42].

Therapies aimed at targeting inflammatory processes in heart failure have produced varied outcomes, thereby raising the question of whether the inflammation observed is a cause or a result of heart failure. The most encouraging findings have emerged from the CANTOS trial (Canakinumab Anti-Inflammatory Thrombosis Outcomes Study, NCT01327846), which illustrated that administration of canakinumab, an IL-1β inhibitor, resulted in a dose-dependent decline in hospitalizations due to heart failure, along with a reduction in a composite endpoint of heart failure hospitalizations and mortality related to heart failure [43]. This evidence suggests a potential therapeutic role for anti-inflammatory treatment in mitigating heart failure progression and improving patient outcomes. It prompts a reconsideration of the inflammatory hypothesis in heart failure, suggesting that specific inflammatory pathways, when appropriately targeted, could offer a viable strategy for disease management. Despite this promise, the translation of these results into clinical practice requires careful consideration of factors such as patient selection, timing of intervention, and cost-effectiveness. Additionally, it highlights the necessity for further research to explore the underlying mechanisms of inflammation in heart failure, identify additional therapeutic targets, and determine the broader applicability of anti-inflammatory strategies in heart failure populations.

## 5. Strengths and Limitations

Our study presents a comprehensive analysis of the prognostic utility of the NLR in patients with acute heart failure, standing out due to its extensive sample size and rigorous methodology. A distinctive methodological advantage of this study is the stratified analysis of patient cohorts based on NLR, employing either cut-off points or quartile comparisons. This approach reduces the risk of bias associated with pooling data from inherently diverse patient populations, thereby improving the accuracy of the results. However, a major limitation of our study was that the results were derived from studies of an observational nature. Observational research designs, while valuable for identifying associations and trends within large datasets, inherently limit our ability to infer causality. While adjusted estimates were used for pooled analysis, the presence of confounding bias could not be entirely ruled out. Furthermore, inconsistencies in the adjustment variables across the included studies might have influenced the outcomes. Variation in study methodologies, such as the stratification of NLR into quartiles or the establishment of specific cut-off points to delineate high- and low-NLR groups, further complicates the analysis. Prior meta-analyses have not adequately accounted for these factors, often treating these diverse approaches as equivalent [13]. Our focused examination of patients with acute heart failure enhances comparability relative to studies encompassing both acute and chronic conditions. The analysis revealed significant heterogeneity among the pooled results, posing challenges to data synthesis and interpretation. Despite efforts to identify the sources of this variability through leave-one-out analyses, specific factors remain to be explored. Factors contributing to heterogeneity likely include disparities in study design (prospective versus retrospective), participant demographics (such as age and gender distribution, along with the prevalence of advanced heart failure), and the criteria for NLR cut-offs. Moreover, we encountered limitations in directly comparing outcomes due to the absence of a universally accepted NLR cut-off value, contributing to inconsistent findings. The limited number of studies addressing certain outcomes further restricted the potential for detailed subgroup analyses or meta-regression to explore the reasons for the observed heterogeneities. A particularly notable observation was the lack of significant difference in in-hospital mortality between patients in the highest versus the lowest NLR quartiles, potentially attributable to the variability in study methodologies and the limited number of studies (n = 3) included this analysis. These limitations underscore the necessity for standardized approaches in future studies to facilitate more definitive conclusions regarding NLR’s role in predicting outcomes in patients with heart failure.

## 6. Conclusions

Our study showed that elevated NLR could serve as a poor prognostic indicator in terms of short- and long-term mortality in patients with acute heart failure. Further large cohort studies, controlling for all important confounders, should be performed to clarify the prognostic role of NLR.

## Figures and Tables

**Table 1:** Baseline characteristics of included studies.

Study	Year	Study Design	Country	Sample Size, n	Age	Female, %	NYHA Class III/IV, %	NLR
Turfan et al., 2014 [16]	2014	Prospective	Turkey	167	67.72 ± 2.29	39.5	74.3	Non-survivors: 7.2 ± 4.8 Survivors: 4.8 ± 3 NLR cut-off: 4.78
Angkananard et al., 2021 [17]	2021	Retrospective	Thailand	321	67.4 ± 14.9	55.1	100.0	Total: 3.7 ± 2.45 NLR cut-off 3.29
Pourafkari et al., 2018 [18]	2018	Retrospective	United States	554	N/A	N/A	N/A	In-hospital mortality Non-survivors: 8 ± 7.5 Survivors: 6.2 ± 4.8 Long-term mortality Non-survivors: 6.5 ± 4.7 Survivors: 5.6 ± 4.2
Turcato et al., 2019 [19]	2019	Retrospective	Italy	439	81.51 ± 1.52	43.7	24.6	Non-survivors: 14 ± 16.1 Survivors: 4.43 ± 2.98 NLR cut-off: 5.70
Huang et al., 2017 [20]	2017	Retrospective	Taiwan	1923	76 ± 12	32.0	N/A	Non-survivors: 6.26 ± 6.80 Survivors: 4.76 ± 5.35
Zhu et al., 2022 [21]	2022	Prospective	China	538	61.1 ± 16.0	33.6	83.1	Non-survivors: 3.47 ± 2.29 Survivors: 2.69 ± 1.79 NLR cut-off 2.28
Ostrowska et al., 2017 [22]	2017	Retrospective	Poland	638	68.3 ± 13.37	34.0	N/A	Non-survivors: 9 ± 8.48 Survivors: 3.73 ± 2.30
Sadeghi et al., 2020 [23]	2020	Retrospective	Iran	197	N/A	38.6	29.4	Non-survivors: 7.61 ± 5.62 Survivors: 3.84 ± 2.82 NLR cut-off: 7.5
Tasal et al., 2014 [24]	2014	Retrospective	Turkey	553	63.4 ± 14.9	33.5	100.0	Non-survivors: 10.2 ± 8.4 Survivors: 6.1 ± 5.3 NLR cut-off: 5.54
Cho et al., 2020 [25]	2020	Prospective	Korea	5580	N/A	46.9	84.9	Quartile 1: 0.2-2.0 Quartile 2: 2.1-3.2 Quartile 3: 3.3-5.8 Quartile 4: 5.9–192.4 NLR cut-off: 5.0
Uthamalingam et al., 2011 [26]	2011	Prospective	United States	1212	73.99 ± 2.25	50.0	70.5	Tertile 1: 2.93 ± 1.19 Tertile 2: 5.13 ± 0.97 Tertile 3: 10.1 ± 4.09
Curran et al., 2021 (GoDARTS) [11]	2021	Retrospective	Multicenter (Europe)	1622	74 ± 10	33.0	57.0	Biostat-CHF cohort—NLR cut-off: 3.22 GoDARTS cohort—NLR cut-off: 4.29
Liu et al., 2023 [12]	2023	Retrospective	China	1169	69.51 ± 13.83	3.6	N/A	Total: 8.43 ± 6.21 Tertile 1: <5.43 Tertile 2: 5.43–10.33 Tertile 3: ≥10.33
Durmus et al., 2015 [27]	2015	Prospective	Turkey	56	67.5 ± 12.6	42.9	N/A	Total cohort: 5.5 ± 2.8 NLR cut-off: 5.1
Tamaki et al., 2023 [28]	2023	Prospective	Japan	1026	82.33 ± 7.42	55.0	N/A	Total cohort: 4.27 ± 2.75 NLR cut-off: 4.5

NYHA: New York Heart Association; NLR: neutrophil-to-lymphocyte ratio; N/A: not available.

## Data Availability

The data supporting this study are included in the article and its Supplementary documents.

## Supplementary Materials

The following supporting information can be downloaded at https://www.mdpi.com/article/10.3390/jcm13051212/s1, Table S1: Newcastle-Ottawa scale for quality assessment and bias assessment of the included studies; Table S2: Covariates Used for Adjustment in Short-Term and Long-Term Mortality; Table S3: Certainty of evidence; Figure S1: The Preferred Reporting Items for Systematic Reviews and Meta-Analyses (PRISMA) flow diagram; Figure S2: Sensitivity analysis for in-hospital mortality (based on high vs low NLR); Figure S3: Funnel plot for in-hospital mortality (based on high vs low NLR); Figure S4: Funnel plot for in-hospital mortality (based on high vs low NLR) after trim-and-fill method; Figure S5: Sensitivity analysis for in-hospital mortality based on highest vs lowest NLR quartiles; Figure S6: Funnel plot for mean differences in NLR between non-survivors and survivors during index hospitalization.
