# Supplementary material for: Prognostic Value of Neutrophil-to-Lymphocyte Ratio in Patients with Acute Decompensated Heart Failure: A Meta-Analysis
Source: J Clin Med. 2024 Feb 21;13(5):1212. doi: 10.3390/jcm13051212 (PMC10931846; doi:10.3390/jcm13051212)
Supplement: Supplementary file 1 [file jcm-13-01212-s001.zip › jcm-2779437-supplementary.pdf]

Supplementary Table 1: Newcastle-Ottawa scale for quality assessment and bias assessment of the included studies

Supplementary Table 2: Covariates Used for Adjustment in Short-Term and Long-Term Mortality

Supplementary Table 3: Certainty of evidence

Supplementary Figure 1: The Preferred Reporting Items for Systematic Reviews and Meta-Analyses (PRISMA) flow diagram

Supplementary Figure 2 Sensitivity analysis for in-hospital mortality (based on high vs low NLR)

Supplementary Figure 3 Funnel plot for in-hospital mortality (based on high vs low NLR)

Supplementary Figure 4 Funnel plot for in-hospital mortality (based on high vs low NLR) after trim-and-fill method

Supplementary Figure 5 Sensitivity analysis for in-hospital mortality based on highest vs lowest NLR quartiles

Supplementary Figure 6 Funnel plot for mean differences in NLR between non-survivors and survivors during index hospitalization

Supplementary Table 1: Newcastle-Ottawa scale for quality assessment and bias assessment of the included studies

**Study**	**Selection**				**Comparability**	**Exposure**			**Total**	**AHRQ standards**
	Representativeness of the exposed cohort	Selection of the non exposed cohort	Ascertainment of exposure	Demonstration that outcome of interest was not present at start of study	Comparability of cohorts on the basis of the design or analysis	Assessment of outcome	Was followed-up long enough for outcomes to occur	Adequacy of follow up of cohorts		
Turfan et al 2014 [16]	★	★	★	★	0	★	★	★	7	poor
Angkananard et al 2021 [17]	★	★	★	★	★	★	★	★	8	fair
Pourafkari et al 2018 [18]	★	★	★	★	0	★	★	★	7	poor
Turcato et al 2019 [19]	★	★	★	★	★★	★	★	★	9	good
Huang et al 2017 [20]	★	★	★	★	0	★	★	★	7	poor
Zhu et al 2022 [21]	★	★	★	★	★★	★	★	★	9	good
Ostrowska et al 2017 [22]	★	★	★	★	0	★	★	★	7	poor
Sadeghi et al 2020 [23]	★	★	★	★	0	★	★	★	7	poor
Tasal et al 2014 [24]	★	★	★	★	0	★	★	★	7	poor
Cho et al 2020 [25]	★	★	★	★	★★	★	★	★	9	good
Uthamalingam et al 2011 [26]	★	★	★	★	★★	★	★	★	9	good
Curran et al 2021 (GoDARTS	★	★	★	★	★★	★	★	★	9	good

) [11]										
Liu et al 2023 [12]	★	★	★	★	★★	★	★	★	9	good
Durmus et al 2015 [27]	★	★	★	★	★★	★	★	★	9	good
Tamaki et al 2023 [28]	★	★	★	★	★★	★	★	★	9	good

Supplementary Table 2: Covariates Used for Adjustment in Short-Term and Long-Term Mortality

**Study**	Covariates used for adjustment in short-term mortality	Covariates used for adjustment in long-term mortality
Turfan et al 2014 [16]	-	-
Angkananard et al 2021 [17]	NYHA classification, initial systolic blood pressure, and initial respiratory rate	
Pourafkari et al 2018 [18]	-	-
Turcato et al 2019 [19]	Age, NYHA class, echocardiography parameters, history of ischemic heart disease, atrial fibrillation, diabetes, and for the values of hemoglobin, creatinine and WBC count	
Huang et al 2017 [20]	-	-
Zhu et al 2022 [21]		Age, sex, MAP, BUN and Log2 NT-proBNP
Ostrowska et al 2017 [22]	-	-
Sadeghi et al 2020 [23]	-	-
Tasal et al 2014 [24]	-	-
Cho et al 2020 [25]	Age category (70> vs. 70≤), sex (male vs. female), body mass index category (25> vs. 23≤), etiology of heart failure (ischemic vs. non-ischemic), systolic blood pressure (100> vs. 100≤), history of hypertension, history of diabetes mellitus, history of cerebrovascular disease, history of chronic obstructive disease, prior admission history due to HF, presented tachyarrhythmia on admission, sodium level (135> vs. 135≤), creatinine level (2.0> vs. 2.0≤), left ventricular ejection fraction (HFrEF vs HFmrEF vs HFpEF), BNP ≥ 500 pg/mL or NT-proBNP ≥1 500 pg/mL	Age category (70> vs. 70≤), sex (male vs. female), body mass index category (23> vs. 23≤), etiology of heart failure (ischemic vs. non-ischemic), systolic blood pressure (100> vs. 100≤), history of hypertension, history of diabetes mellitus, history of cerebrovascular disease, history of chronic obstructive disease, prior admission history due to HF, presented tachyarrhythmia on admission, sodium level (135> vs. 135≤), creatinine level (2.0> vs. 2.0≤), left ventricular ejection fraction (HFrEF vs. HFmrEF vs. HFpEF), BNP ≥ 500 pg/mL or NT-proBNP ≥ 1500 pg/mL, AA, BB, RASi.

Uthamalingam et al 2011 [26]	-	Age, gender, cardiac risk factors, the LVEF, medications, hemoglobin, blood urea nitrogen, creatinine, B-type natriuretic peptide, and atrial fibrillation
Curran et al 2021 (GoDARTS) [11]	-	Age, HF hospitalization in the previous year, peripheral oedema, systolic blood pressure, estimated glomerular filtration rate, urea, N-terminal pro-brain natriuretic peptide, haemoglobin, high-density lipoprotein cholesterol, sodium and beta-blocker use
Liu et al 2023 [12]	-	Age, SBP, DBP, HR, RH, AF, AMI, respiratory failure, VF, platelet, BUN, creatinine, glucose, NT-proBNP, diuretics, vasopressin, length of ICU stay, SOFA score, SAPS II
Durmus et al 2015 [27]	-	Age, sex, EF, PLR
Tamaki et al 2023 [28]	-	Age, sex, hypertension, diabetes, coronary artery disease, hemoglobin, estimated glomerular filtration rate, and N-terminal pro-B-type natriuretic peptide level.

Supplementary Table 3: Certainty of evidence

No of studies	Design	Risk of bias	Inconsistency	Indirectness	Imprecision	Other	Certainty (overall score)
**Outcome: Mortality (High vs Low NLR)**							
8	Observational studies	not serious	serious	not serious	serious	none	⊕⊖⊖⊖ very low
**Outcome: Mortality (Highest tertile quartile vs lowest tertile/quartile)**							
4	Observational studies	not serious	serious	not serious	serious	none	⊕⊖⊖⊖ very low
**Outcome: NLR mean difference**							
8	Observational studies	not serious	serious	not serious	serious	none	⊕⊖⊖⊖ very low

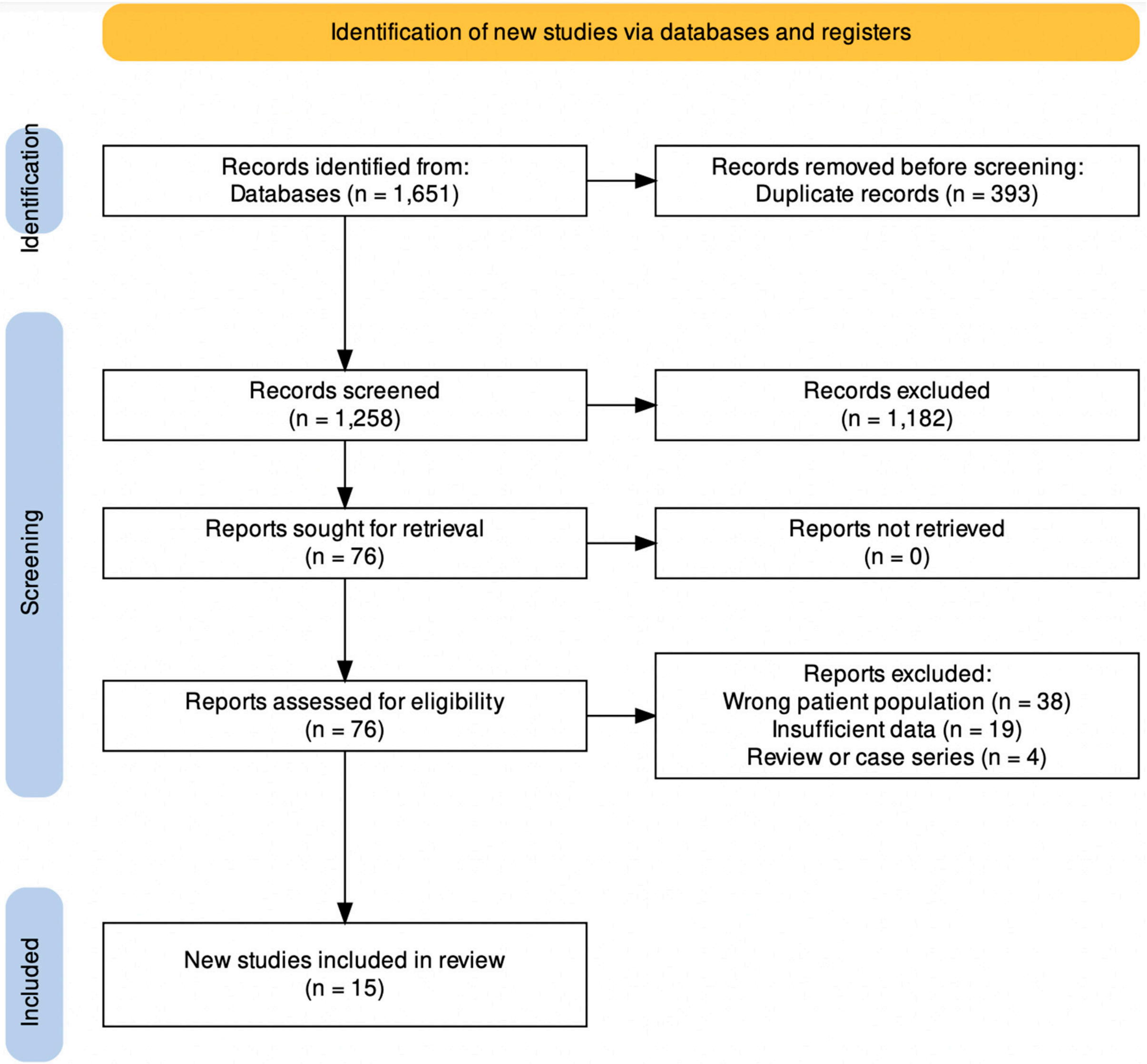

Supplementary Figure 1: The Preferred Reporting Items for Systematic Reviews and Meta-Analyses (PRISMA) flow diagram

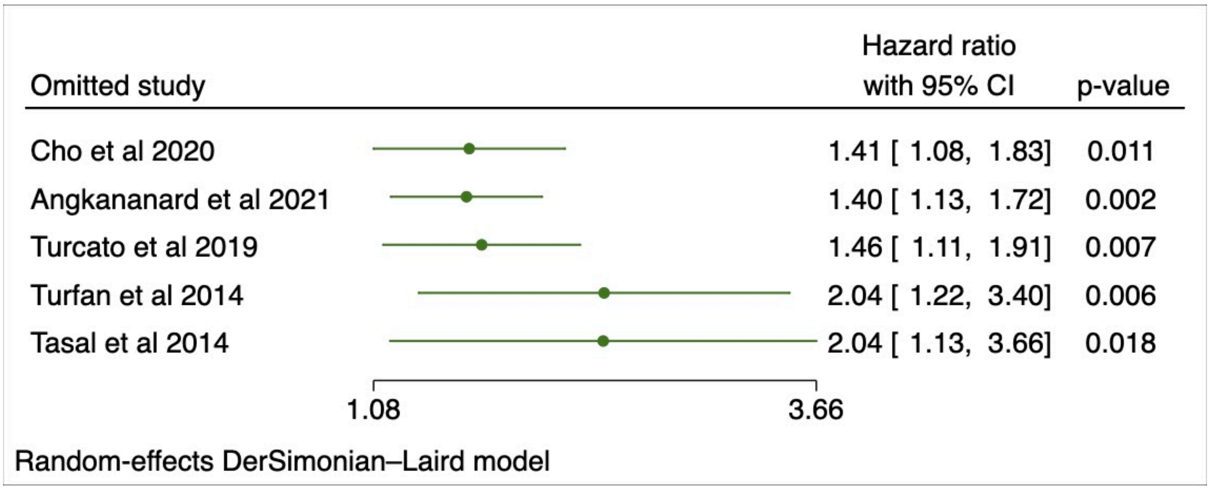

Supplementary Figure 2 Sensitivity analysis for in-hospital mortality (based on high vs low NLR)

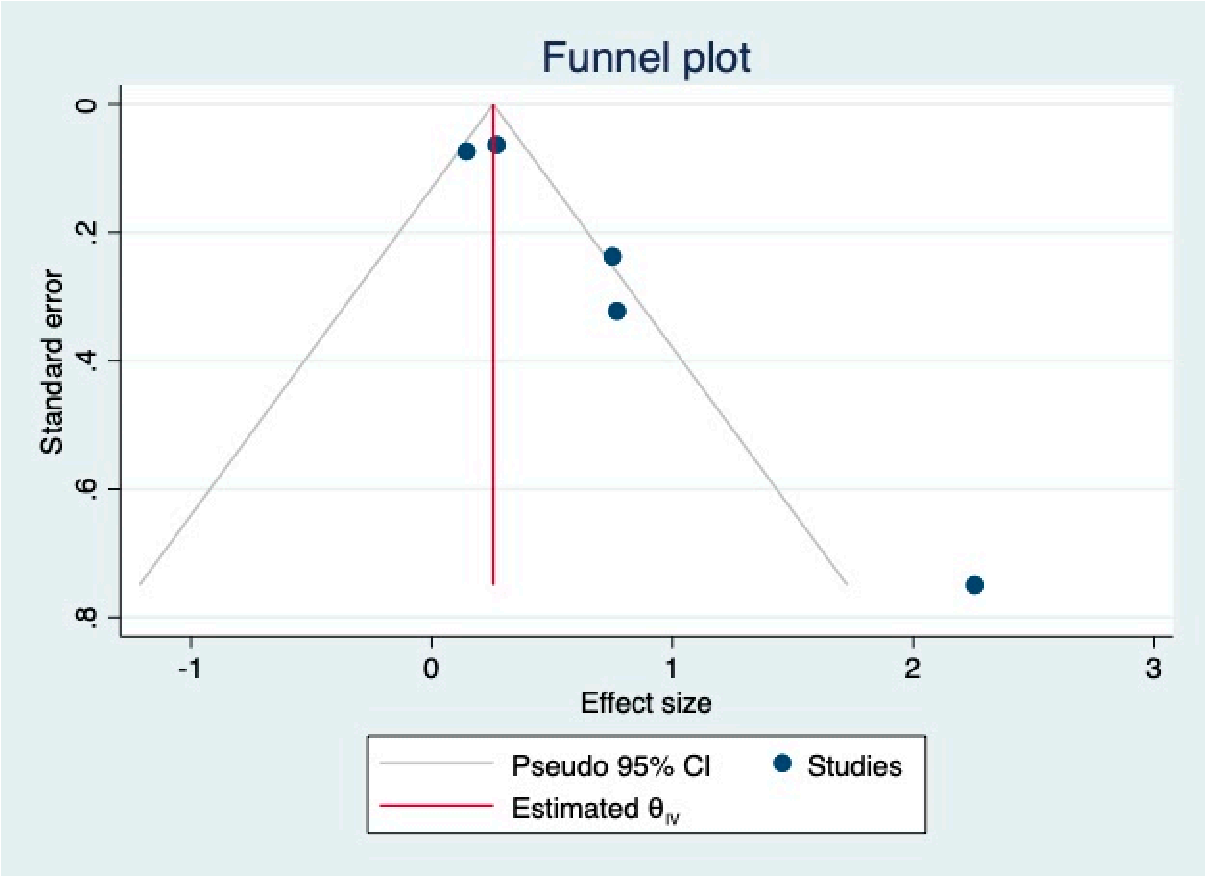

Supplementary Figure 3 Funnel plot for in-hospital mortality (based on high vs low NLR)

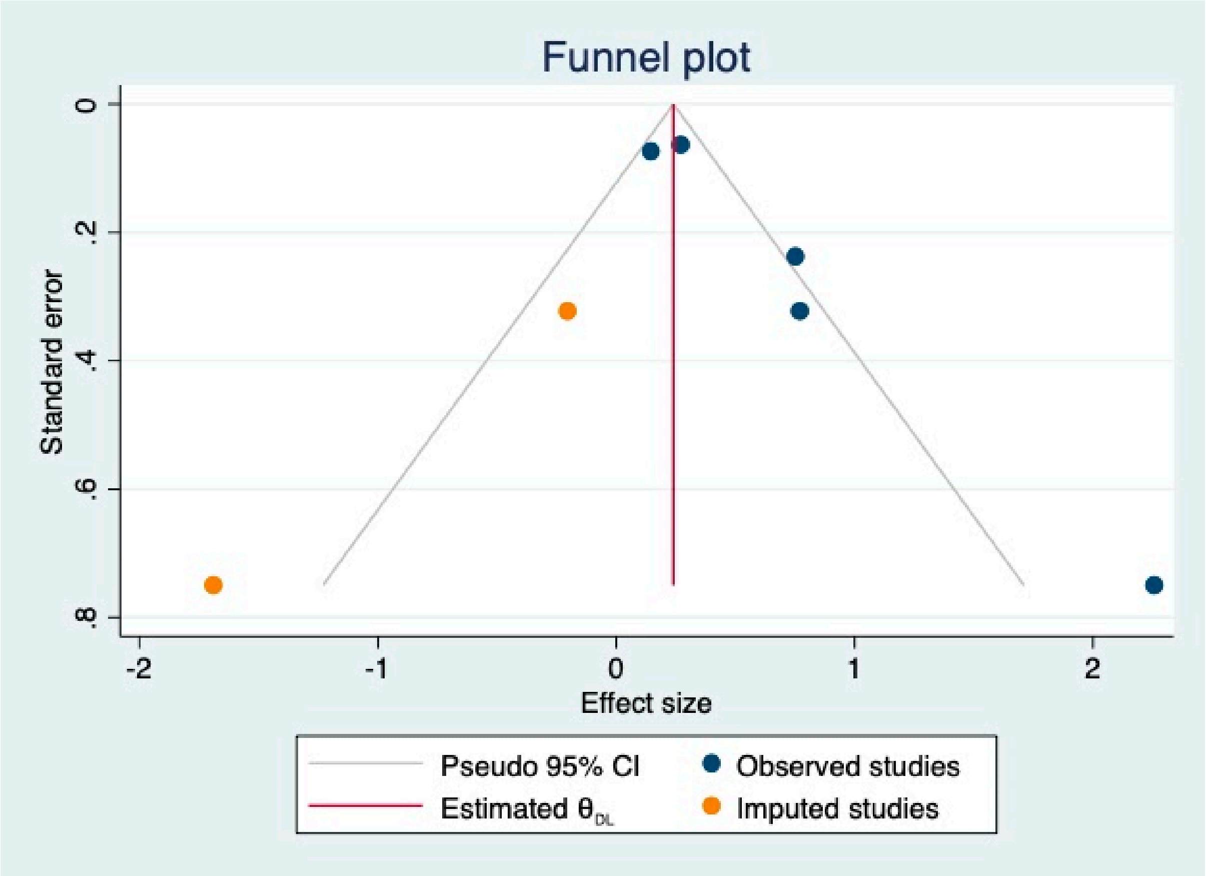

Supplementary Figure 4 Funnel plot for in-hospital mortality (based on high vs low NLR) after trim-and-fill method

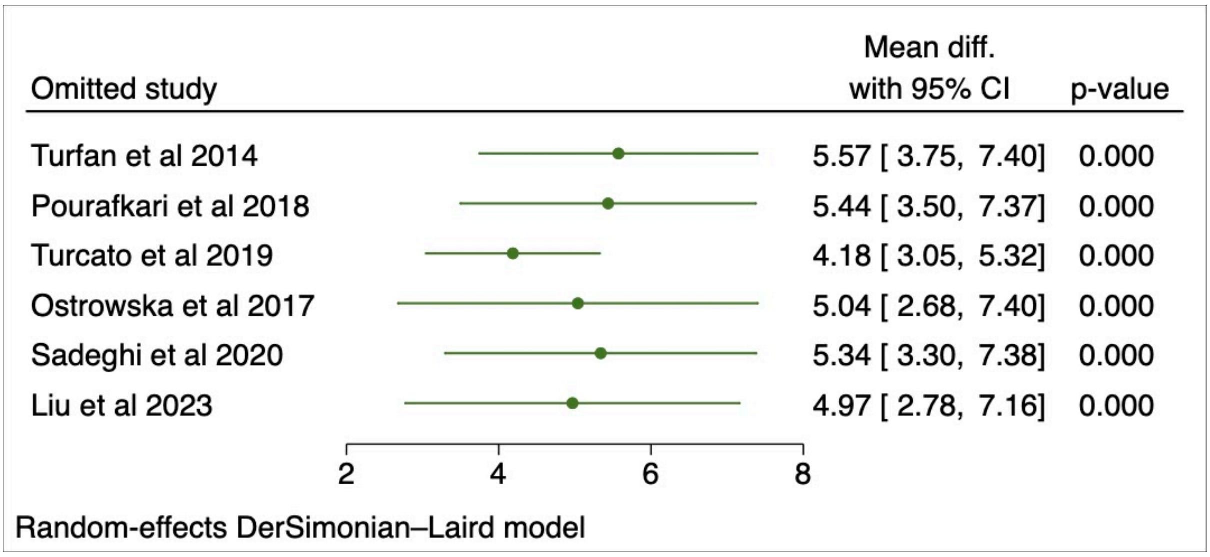

Supplementary Figure 5 Sensitivity analysis for mean differences in NLR between non-survivors and survivors during index hospitalization

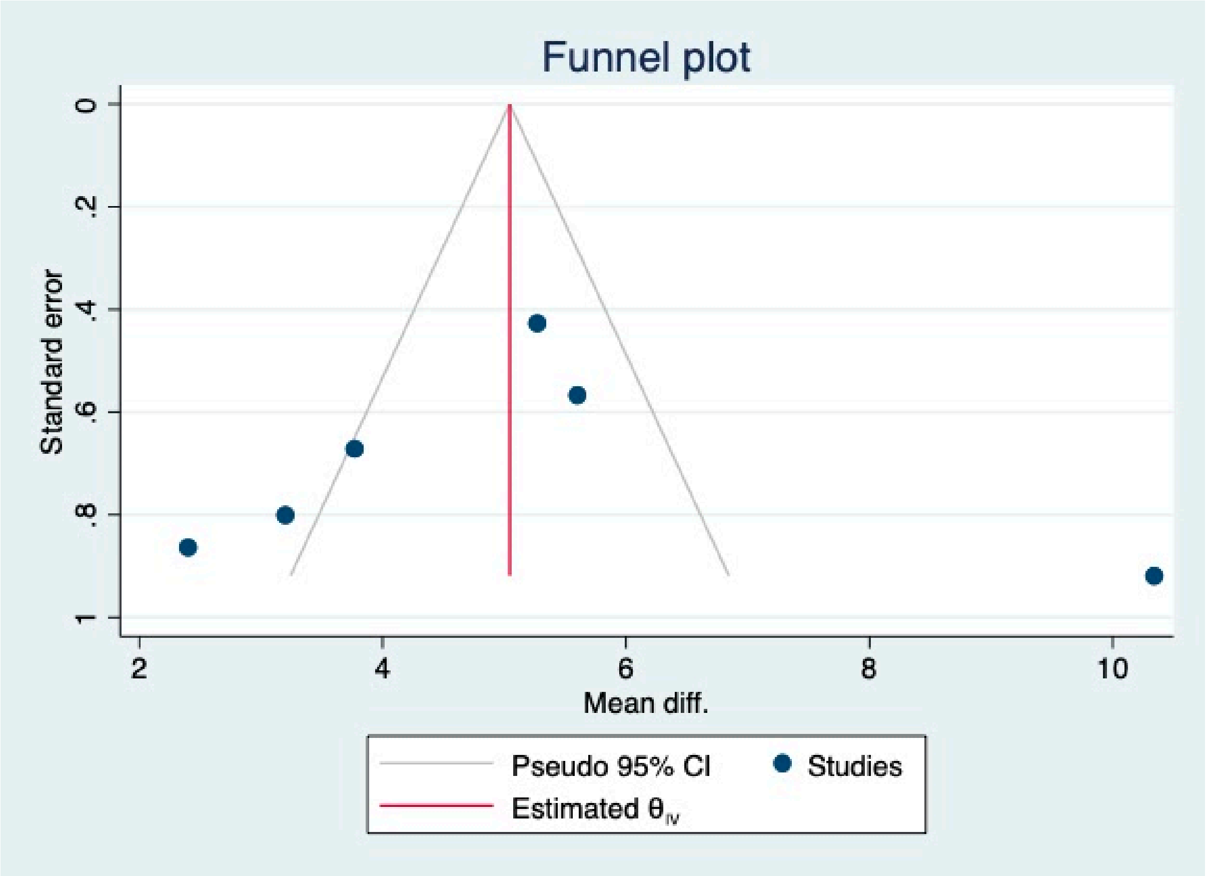

Supplementary Figure 6 Funnel plot for mean differences in NLR between non-survivors and survivors during index hospitalization
